# TET1 mitigates prenatal fluoride-induced cognition impairment by modulating *Bcl2* DNA hydroxymethylation level

**DOI:** 10.1186/s10020-025-01174-w

**Published:** 2025-03-25

**Authors:** Yongle Cai, Xingdong Zeng, Mengyan Wu, Haonan Chen, Miao Sun, Hao Yang

**Affiliations:** 1 Institute for Fetology, The First Affiliated Hospital of Soochow University, Suzhou, Jiangsu 215006 China; 2 School of Basic Medical Sciences, Ningxia Medical University, Yinchuan, Ningxia 750004 China

**Keywords:** Fluoride, Cognition deficits, Neuron apoptosis, TET1, *Bcl2*, Hydroxymethylation

## Abstract

Fluoride exposure during pregnancy commonly compromises fetal neurodevelopment and largely results in a broad spectrum of cognitive deficiencies in the adult offspring. However, the precise mechanisms underlying these effects remain to be fully elucidated. Herein, we investigate the impacts of fluoride on neural excitability and apoptosis, synaptic plasticity, and cognitive function, as well as possible underlying mechanisms. Our results indicated that exposure to a high sodium fluoride (100 mg/L) during pregnancy in the mouse can cause the cognitive deficits of their offspring, accompanied by a decrease in the expression of Tet-eleven translocation protein 1 (TET1), an enzyme responsible for DNA hydroxymethylation. Additionally, there is a reduction in the dendritic spine density and the expression of postsynaptic density protein-95 (PSD95) in the hippocampal regions of male offspring. Furthermore, in vitro fluoride treatment significantly exacerbates neuronal apoptosis and reduces the frequency of spikes in spontaneous action potential. More significantly, we also found that TET1 could directly bind to the promotor region of *Bcl2*, altering its DNA hydroxymethylation and *Bcl2* expression. Intriguingly, *Tet1* knock-out mice exhibited cognitive deficits similar to those observed in male animals exposed to high levels of fluoride. Furthermore, the down-regulation of TET1 protein, along with the consequent alteration in *Bcl2* hydroxymethylation and increased neuronal apoptosis, are likely mechanisms underlying the impact of prenatal fluoride exposure on the neurodevelopment of male offspring. These findings provide novel insights into the molecular mechanisms by which fluoride exposure induces neurodevelopmental impairment of the male offspring.

**Supplementary Information:**

The online version contains supplementary material available at 10.1186/s10020-025-01174-w.

## Introduction

Fluoride is a chemically reactive element widely distributed in the environment at a variety of concentrations. Although fluoride at appropriate doses has been employed to prevent osteoporosis and dental cavities, excessive amounts of fluoride ions in drinking water can induce skeletal fluorosis, dental fluorosis, fatigue, and impaired cognitive neurodevelopment (Rahmani et al. 2023; Solanki et al. 2022; Veneri et al. 2023; Whelton et al. 2019). More notably, chronic exposure to concentration of fluoride in animals has been identified as a critical risk factor for anxiety-depressive symptoms, cognition abnormality, autism spectrum disorder and other dysfunctions, as evidenced by a multitude of relevant studies (Adkins and Brunst 2021; Strunecka and Strunecky 2019). The excess accumulation of fluorides in the cerebrum can seriously impair children’s neurodevelopment, primarily due to the blood-brain barrier’s limited ability during this developmental period to effectively prevent fluoride from entering the neurological system (Zheng et al. 2003; Zwierello et al. 2023). Up until now, the majority of studies have focused on the detrimental effects of fluoride on both children and adults, revealing that fluoride readily enters the fetus through placenta, thereby significantly impacting the early development of the central nervous system (CNS). However, very little is understood regarding the effects and underlying mechanisms of fluoride on brain growth and development in the fetuses and neonates. It is well-known that normal neurogenesis and the functional construction of neural circuits during the early stages of neurodevelopment are crucial for neurobehavioral performance in both the postnatal and adult periods (Wamsley and Fishell 2017). Given that neurons are highly polarized cells with two structurally and functionally distinct compartments, such as cell body, neurites and synapses, the regulation of neuronal excitability and synaptic transmission via various ion channels is fundamental to motor and cognitive behavior, as well as synaptic function (Ancaten-Gonzalez et al. 2023).

Recent studies have indicated that NaF exacerbates oxidative stress and mitochondrial dysfunction while concurrently inhibiting autophagic flux in hippocampal neurons, ultimately resulting in neuronal damage in murine models. It has been documented that honokiol enhances SOD2 activity and expression, thereby reducing mtROS through Sirt3 activation, which ameliorates cognitive deficits (Ran et al. 2023; Wang et al. 2022). Furthermore, some researchers propose that the disruption of mitochondrial homeostasis plays a pivotal role in NaF-induced neurotoxicity. Their investigations have demonstrated that resveratrol exerts neuroprotective effects by activating SIRT1, enhancing mitochondrial biogenesis and function, and thereby attenuating NaF-induced neurotoxicity (Zhao et al. 2020). Notably, NaF has also been shown to disrupt calcium homeostasis in neutrophils, leading to the activation of L-type calcium ion channels (LTCCs). This activation facilitates the influx of free iron into neutrophils via LTCCs, ultimately triggering neutrophil ferroptosis. The subsequent formation of neutrophil extracellular traps (NETs) exacerbates neuroinflammation, thereby contributing to brain damage (Wang et al. 2023). The findings indicate that NaF has the potential to harm the nervous system through various mechanisms. Nevertheless, the precise manner in which NaF exposure during embryonic development results in cognitive impairment in adulthood remains a critical and unresolved issue.

Epigenetic modifications, particularly DNA methylation and the subsequent chromatin remodeling, plays a critical role in the maintenance and differentiation of embryonic stem cells (ESCs) as well as for the regulation of early embryo development (Wang et al. 2021). Aberrant epigenetic modifications can elevate the risk of birth defects, infertility and congenital anomalies in offspring. Unfortunately, a growing number of studies indicated that fluoride exposure may cause epigenetic alterations during critical stages of mammalian development, as evidenced by both in vitro and in vivo (Balasubramanian and Perumal 2022). Although fluoride has been linked to various neurological disorders due to its epigenetic toxicity on the development and maturation of neural circuits, as well as the neuronal electrophysiology, the specific epigenetic alterations within the developing CNS remain unclear. The TET1 protein, a DNA hydroxymethylation enzyme, functions as a tissue-specific and temporal regulator of gene expression, playing a crucial role in linking between environmental factors to gene expression (Armstrong et al. 2023). The dynamic regulation of epigenetic modifications in DNA is vital for processes such as long-term memory consolidation, synaptic transmission, neuronal activation, and adaptive responses in neurons. Consequently, the TET1 enzyme is essential for proper brain function, and mutations or variations in TET1 expression may be implicated in, or even primarily responsible for, cognitive deficits in mammals (Greer et al. 2021). PSD95, a highly abundant postsynaptic scaffolding protein in excitatory synapses, plays an essential part in synaptogenesis and dendritic spine morphogenesis during neurodevelopment, as well as for synaptic plasticity and cognitive functions such as learning and working memory. This is achieved through its organization and interaction with various subtypes of neurotransmitter receptors, including AMPA and NMDA receptors (Bulovaite et al. 2022). The potential impact of prenatal fluoride exposure on synaptic plasticity in adult offspring, and the role that *Tet1* may play in the underlying mechanisms, has yet to be investigated.

In this study, we employed a prenatal fluoride exposure mouse model, a *Tet1* knockout model, and primary neuron culture assays to comprehensively investigate the reciprocal regulation of *Tet1* and *Bcl2* in relation to hippocampal neuron functions and apoptosis. In addition, the impact of epigenetic modifications, particularly DNA hydroxymethylation, on cognitive function in male mice subjected to prenatal fluoride were investigated. Our results indicate that TET1 mitigates prenatal fluoride-induced cognitive impairment, at least in part, by modulating *Bcl2* DNA hydroxymethylation and the consequently reducing neuron apoptosis. The present study could provide a basis for further investigation of the molecular mechanism by which TET1 attenuate fluoride exposure-induced neurodevelopmental impairments, while also seeking a potential therapeutic target for the treatment of this neurodevelopment disorder.

## Materials and methods

### Animals and treatment

All experimental procedures and protocols used in this study were approved by Animal Experimentation Ethics Committee for use of experimental animals at the first affiliated hospital of Soochow University. Adult male and female C57/BL6J mice aged 8 weeks were used for all experiments. The animals (specific pathogen-free) were housed at constant temperature (22 °C to 24 °C) and humidity (40–60%) on a 12 h light/dark cycle with constant air renewal. All mice had free access to standard food and water. Notably, mice aged 8 weeks were randomly assigned each group and continuously housed in cages with a male to female ratio of 1:2. Once a vaginal plug-like formation was found at females in the morning, the day was recorded as gestational age 0 day. Subsequently, the pregnant female mice were administered orally to NaF in drinking water at the concentration of 100 mg/L. Twenty-one days later, the drinking water with 100 mg/L was replaced by normal drinking water when the offspring were born. The male and female mice were isolated in individual cages until weaning at 21 days of age.

### Mouse genotyping

When offspring are weaned at 21 days of age, the 2–3 mm of mice’s tail snips were obtained, and introduced into 100 µL Beyotime’s DNA extraction solution containing an enzyme mix at 55 °C for 15 min and 95 °C for 5 min, respectively. Concomitantly, a stop solution was added to terminate DNA digestion extracted from tail snips. For PCR reactions, a mixture of 2 µL DNA lysates, 2 µL primers, and 2× DreamTaq Green PCR was prepared. The primers used were TTAAAGCATGGGTGGGAGTC and AACTGATTCCCTTCGTGCAG. Selective amplification via PCR was conducted over 35 cycles, with annealing each time at 55 °C during each cycle. The PCR products then were assayed using 1% agarose gel electrophoresis in a continuous Tris–acetate–EDTA buffer system, and the resulting bands were photographed for analysis. The gene-modified mice were procured from Jackson Laboratory (Stock No. 017358) and genotyping primers were selected according to the supplier’s official protocol. The specificity of these primers was validated through agarose gel electrophoresis. In wild-type (WT) mice, only a 900 bp wild-type band was observed, whereas in knockout (KO) mice, a 650 bp band was detected due to the partial deletion of the target gene. Heterozygous (Het) mice exhibited both bands (Figure S1B).

### Behavioral assessments

#### Morris water maze

To evaluate fluoride-induced impairment of spatial learning and memory, we conducted the Morris water maze test using a circular tank with a diameter of 120 cm and a height of 60 cm, filled with water containing titanium dioxide at 22 °C. In brief, the experiment endured for six days including the positioning navigation trial (PNT) from day 1 to day 5, and the space probe trial (SPT) on day 6. During the PNT phase, eight-week-old male mice from various groups were subjected to four quadrants per day to locate the submerged platform. In the SPT phase, the platform was removed and each mouse was allowed to swim freely in the circular tank for one min.

#### Novel object recognition test and object location behavioral testing

Next, the novel object recognition test (NOR) was conducted to evaluate the mice short-term recognition and memory as described previously (Lu et al. 2024). In brief, the nine-week-old male mice were placed in a test arena (40 cm×40 cm×40 cm) containing two identical cones, and allowed to roam about for 5 min. Concomitantly, the mice were reintroduced to the same arena next day with the familiar cone and a novel cylinder in the identical positions. The total time spent by mice in each quadrant was recorded. The Object Location Behavioral Testing (OLT), similar to the NOR test, differs primarily in that a familiar object is relocated to a new position after one day, specifically to assess the spatial learning ability of the mice. Notably, the recognition index, which is positively correlated with recognition and memory, was calculated using the formula: time spent with novel objects (s) / (time spent with novel objects (s) + time spent with old objects (s).

#### Y-maze test

The Y-maze test was also carried out to assess the short-term memory of nine-week-old male mice. The Y-maze apparatus consists of three identical arms which are set at an angle of 120° angles relative to each other. On the initial day, the mice were permitted to explore the maze freely for a duration of 5 min, followed by a reintroduction into the maze the subsequent day. Lastly, the number of total entrances and alternations were recorded. The spontaneous alternation index was calculated as the ratio of the number of alternations to the total number of entries minus two.

#### Passive avoidance test

For the passive avoidance test, each ten-week-old male mouse was firstly placed into a passive avoidance apparatus equipped with an intelligent compartment with the guillotine door closed for the first training. Ten seconds later, the door was opened, and most of the mice tended to explore the dark compartment. Once the mice entered into the dark compartment, the door was closed, and a mild electrical foot shock was administered, immediately followed by the mice’s fleeing behavior. The foot shock stimulus was delivered at a current of 0.4 mA, a voltage of 40 V, and a duration of 1 s. 24 h later, the test session was conducted. Each mouse was reintroduced to the two compartments with the door open, but without any electrical foot shock. The frequency and latencies of the mice’s transitions from the light to the dark compartment were recorded for a maximum of 300 s.

#### Culture of HT22 cells and primary hippocampal neurons

HT22 cells were purchased from Pricella (Cat. No. CL-0697) and subsequently cultured using Pricella’s specialized HT22 cell culture medium (Cat. No. CM-0697). Once the cells reached near confluence, the cells were passaged via trypsin digestion. All cell cultures were maintained in a 37 °C incubator with 5% CO_2_. Primary neurons were prepared from the hippocampus of both NaF-treated with and normal mice at 14 days of pregnancy as described previously (Maday and Holzbaur 2016). Briefly, pregnant adult female mice were euthanized via cervical dislocation, and their skin was sterilized with 75% ethanol. The embryos were then rapidly and aseptically dissected and collected in PBS solution. Under a stereoscopic microscope, the intact coronal structure of the brain tissue in E14.5 mouse embryos was identified. Following the removal excess tissues, the bilateral cerebral hemispheres were carefully isolated and bisected along the midline. The hippocampal tissue was located posterior to the midbrain and thalamus and was meticulously dissected. The isolated hippocampal tissue was collected in a sterile centrifuge tube, washed three times with PBS, finely was dissected and enzymatically dissociated in 0.125% Trypsin-EDTA at 37 °C for 25 min. After gently mechanical trituration to produce a cell suspension, the dissociated cells were then collected by centrifugation and subsequently diluted to an appropriate cell density. Finally, the cells were seeded into the plastic tissue culture-treated dishes coated with Poly-L-Lysine (PLL) at a density of 2 × 10^5^ cells/cm^2^ in Neurobasal medium supplemented with 2% B27, 0.5mM GlutaMAX, and 1×Penicillin and Streptomycin. The cultures were maintained at 37 °C in a humidified atmosphere of 5% CO_2_ incubator. The culture medium was refreshed once every 3 days. Four days later, the cells treated with normal medium and 0.2mM NaF were collected for further experiments.

### RT-PCR and q-PCR analysis

Total RNA was extracted from mouse hippocampus and cells undergoing the abovementioned different treatments using Trizol reagent according to the manufacturer’s instructions. One microgram of total RNA was reversely transcribed into cDNA by using ThermoFisher RevertAid RT Kit in accordance with the manufacture’s protocol. Fifty nanograms of cDNA was used for PCR amplification. Quantitative RT‒PCR was performed in three replicates of each sample and repeated 3 times. Notably, the genes of interest were *Tet1* and *Bcl2*, and the housekeeping gene *Gapdh* was used as an internal control to normalize the PCR. The SYBR Green-based quantitative real-time PCR (Takara) was used to quantify the mRNA levels. Information regarding the sequences of gene-specific primers mentioned above is provided in Table S1.

### Immunofluorescence

For immunostaining of each treatment group, all cultures on coverslips were washed three times with PBS, followed by fixation with 4% paraformaldehyde (PFA) for 15 min at 4 °C. Subsequently, the cells on coverslips were permeabilized in 0.1% Triton X-100 for 10 min at room temperature (RT), blocked in 5% goat serum for 1 h at RT and incubated with primary antibodies diluted in 1% BSA in PBS overnight at 4 °C. The following primary antibodies were used: rabbit anti-TET1 (1:200), rabbit anti-BCL2 (1:400), mouse anti-TUJ1 (1:100), and rabbit anti-CC3(1:400). Following removal of primary antibodies, the samples were thoroughly rinsed with PBS three times for 5 min. Subsequently, secondary antibodies (ThermoFisher Alexa Fluor 488/594 anti-rabbit IgG and anti-mouse IgG) and DAPI diluted in PBS were incubated for 1 h at RT in the dark. Finally, the coverslips were mounted onto glass slides using an anti-fading mounting medium suitable for microscopy.

### Western blots

To elucidate the signaling molecules involved in mediating cognitive impairment, the hippocampal tissue from different treatment groups was lysed to extract total protein in RIPA buffer supplemented with PMSF or protease inhibitors for 30 min on ice. The protein concentrations of the clarified lysates were determined using a Detergent Compatible Bradford Protein Assay Kit (Beyotime). Subsequently, the supernatant was collected for western blot analysis. In brief, the protein samples were separated on 7.5-15% gradient SDS-PAGE gels and then transferred onto PVDF membranes. The membranes were blocked with 5% fat-free milk in Tris-buffered saline for 1 h at RT. Following blocking, the PVDF membranes were washed three times for 10 min each and incubated with primary antibodies at 4 °C overnight. The primary antibodies used were TET1 (1:2500), BCL2 (1:1000), PSD95 (1:2000), cleaved Caspase-3 (1:1000), and Tubulin (1:1000), with Tubulin serving as the internal loading control. After three washes with TBST, the membranes were incubated with a horseradish peroxidase-conjugated secondary antibody (ThermoFisher, 1:5000) diluted in TBST at RT for 1 h. Following thorough washing in TBST, the immunoblots were detected using enhanced chemiluminescence (ECL).

### Flow cytometry

To investigate neuron survival following sodium fluoride (NaF) treatment over varying durations, a FITC Annexin V Apoptosis Detection Kit with Propidium Iodide (PI) from BioLegend was employed to assess cell apoptosis progression via flow cytometry, adhering to the manufacturer’s protocol. Briefly, after NaF treatment, HT22 cells—a neuronal cell line derived from the hippocampus were detached from the culture plate using 0.125% trypsin, dissociated into single-cell suspensions, washed with cold PBS, and filtered through an 80-µm nylon mesh prior to analysis. The cells were subsequently resuspended in Annexin V Binding Buffer at a concentration of 5 × 10^6^ cells/mL following two washes with cold BioLegend Cell Staining Buffer. Concomitantly, 10 µL of Propidium Iodide Solution and 5 µL of FITC Annexin V were added to the 100 µL cell suspension. After 15 min, 400 µL of Annexin V Binding Buffer was added for flow cytometry analysis. All samples were analyzed by cytometry within 30 min, and data analysis was conducted using FlowJo software.

### Electrophysiology

Four days post-treatment, primary neurons treated with or without 0.2mM NaF were cultured in normal culture medium. These neurons were subsequently subcultured upon reaching confluency. For neuron electrophysiological analysis, the neurons on the coverslip were transferred into artificial cerebrospinal solution (aCSF in mM: 125 NaCl, 25 NaHCO3, 3 KCl, 1.25 NaH_2_PO_4_, 1 CaCl_2_, 6 MgCl_2_ and 25 glucose, PH adjusted to 7.35 with HCl) bubbled with a gas mixture of 95% O_2_ and 5% CO_2_ at 37℃ for 15 min, then maintained at RT. Subsequently, whole-cell action potential was captured with the whole-cell patch-clamp (Axon Multiclamp 700B with Clampex 10.2). The electrode resistances of somatic pipettes were 3–5 MΩ when filled with internal solution (in mM): 135 K-gluconate, 10 Na-phosphocreatine, 1 EGTA, 10 HEPES, 2Na2-ATP, 0.3 Na-GTP, and 2 MgCl_2_, pH adjusted to 7.35 with KOH. After switching to current-clamp mode, the stimulation current was incrementally increased from − 100 pA to 200 pA over a 1-second duration, and the corresponding action potentials were recorded (Figure S1D). Additionally, spontaneous action potentials were recorded over a 10-second interval without the application of current stimulation.

### Nissl staining and Golgi staining

For examination of neuron Nissl substance changes of hippocampal neurons in eight-week-old male mice deficient in NaF and *Tet1*, frozen sections were fixed with 4% PFA for 10 min, rinsed twice in DEPC-treated water for 2 min each, immersed in Nissl staining solutions (Beyotime) for 5 min, and dehydrated using 95% alcohols (2 min each) and xylenes (5 min each). To elucidate the dendrite morphology of hippocampal neurons, the whole brain from each animal was processed for individual neuron impregnation using the FD Rapid GolgiStain™ Kit, following the manufacturer’s instructions, and then coronally sectioned at a thickness of 200 μm.

### CCK8 assay

Neurons from distinct groups were independently seeded into corresponding 96-well plates, with each well containing 200 µL of culture medium. Following a four-day incubation period, 20 µL of CCK-8 solution was introduced to each well. Wells containing solely culture medium and CCK-8 solution, devoid of cells, served as the blank control. Subsequent to an additional two-hour incubation, absorbance was recorded at 450 nm using a microplate reader. Cell viability was calculated using the formula: (Absorbance of experimental wells-Absorbance of blank wells)/ (Absorbance of control wells-Absorbance of blank wells).

### hMeDIP and ChIP-qPCR

The hMeDIP results were obtained using the EpiQuik Hydroxymethylated DNA Immunoprecipitation (hMeDIP) Kit (Cat. No. P-1038). ChIP-qPCR data were obtained from the SimpleChIP^®^ Plus Enzymatic Chromatin IP Kit (Magnetic Beads) from Cell Signaling Technology (Cat. No. 9005). All experimental procedures involving these kits were strictly conducted in accordance with the manufacturer’s protocols.

### Statistics

All data are expressed as mean ± SEM. Statistical significance between groups was determined by unpaired t-test. Shapiro-Wilk test was employed to assess the normality for all the data. When data failed to pass the normality test, Mann-Whitney and Kruska-Wallis tests were used as non-parametric equivalents. A p-value < 0.05 was considered statistically significant.

## Results

### The effect of prenatal fluoride exposure on cognition in male offspring mice

**Fig. 1:**
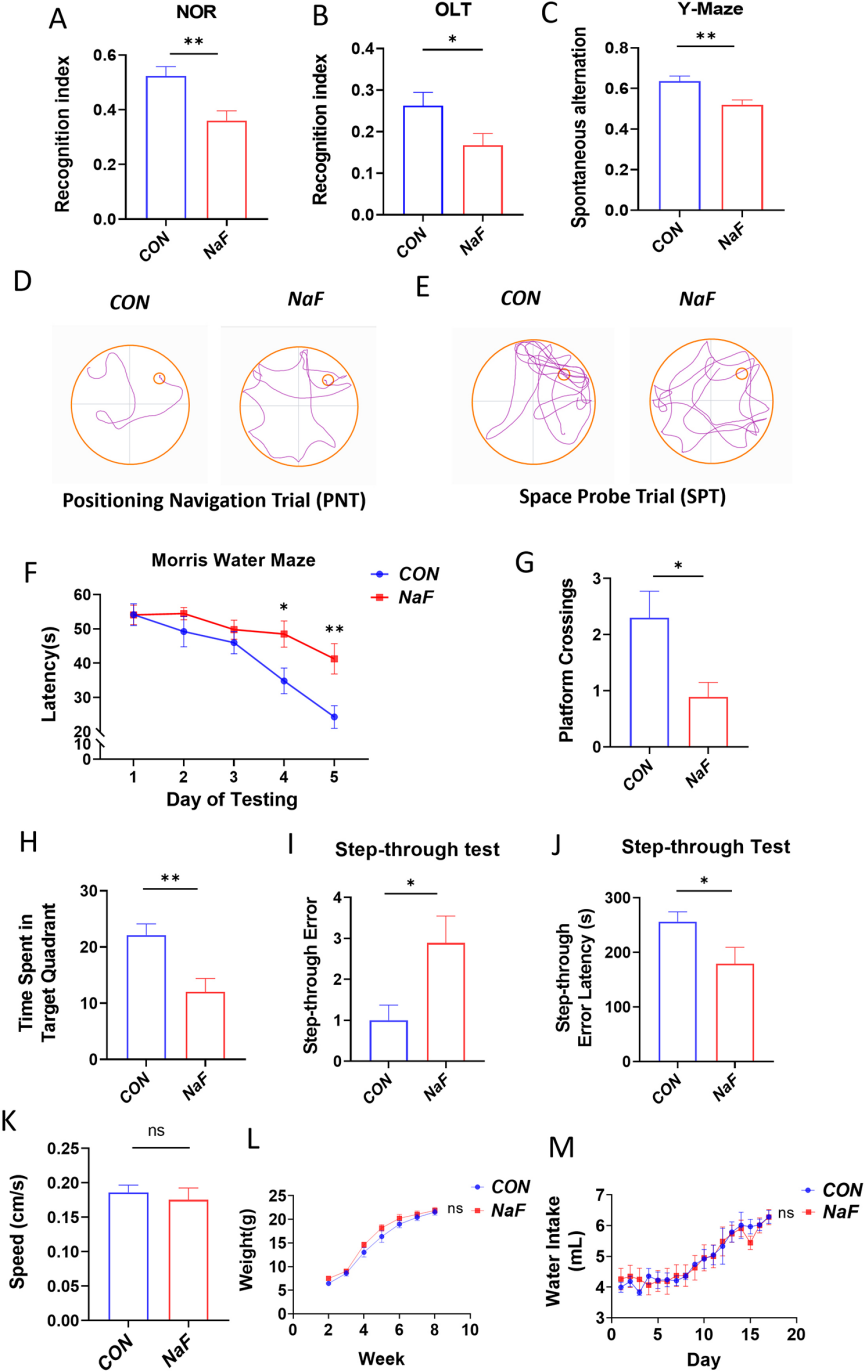
Cognitive assessments of NaF-exposed mice offspring (**A**) Novel object recognition (NOR). (**B**) Object location behavioral testing (OLT). (**C**) Y-Maze test (*n* = 19). (**D**, **E**) Representative pathway maps of positioning navigation trail (PNT) and space probe trail (SPT) in Morris water maze test. (**F**) Escape latency of NaF-exposed adult mouse offspring with hidden platform (*n* = 19). (**G**) Number of Platform crossings by NaF-exposed adult mouse offspring in Morris water maze test (*n* = 19). (**H**) Time spent in the target quadrant of NaF-exposed adult mouse offspring during the SPT (*n* = 19). (**I** and **J**) Error frequency and the duration before initial entry into the dark chamber during the passive avoidance test for NaF-exposed adult mouse offspring (*n* = 19). (**K**) Examination of swimming speed of NaF-exposed mice. Notably, no significant difference was observed between two groups in Morris water maze test (*n* = 19). (**L** and **M**) Changes in offspring weight (*n* = 21) and maternal water intake during gestation under the indicated treatments (*n* = 11). All data are reported as means ± SEM. Statistical significance is indicated by *p < 0.05 and **p < 0.01, in comparison to the respective control groups

To validate the potential impact of prenatal fluoride exposure on neurocognitive development, spatial learning and memory in mice were assessed using the Morris water maze test, novel object recognition test (NOR), Y maze, and passive avoidance test. As shown in Fig. 1, offspring mice exposed to 100 mg/L NaF during specified periods of gestation (NaF mice) exhibited cognitive impairments compared to control animals. Notably, the NaF mice demonstrated a significant decline in the recognition index in both the NOR and OLT tests, as well as in spontaneous alternation behavior, relative to the control group (Fig. 1A, B, and C). Consistent with these findings, the Morris water maze test showed a marked reduction in spatial learning and memory in NaF-exposed animals in both the PNT and SPT (Fig. 1D and E). Specifically, NaF mice required significantly more time to locate the hidden platform and exhibited a reduced frequency of platform crossings and decreased time spent in the target quadrant compared to sham-treated mice (Fig. 1F, G, and H). In the subsequent probe test, the NaF mice displayed a higher number of step-through errors and a reduced latency in step-through errors during the passive avoidance experiment compared to the sham mice (Fig. 1I and J). Nonetheless, the NaF mice demonstrated swimming speeds comparable to those of the control mice, indicating that NaF (100 mg/L) fails to impair the motor system (Fig. 1K). Furthermore, no significant difference was found in offspring weight and water intake during pregnancy between NaF mice and the control mice (Fig. 1L and M). Collectively, the data above suggest the prenatal fluoride exposure may cause cognitive and spatial memory impairment in offspring.

### Neurons require TET1 protein against apoptosis

**Fig. 2:**
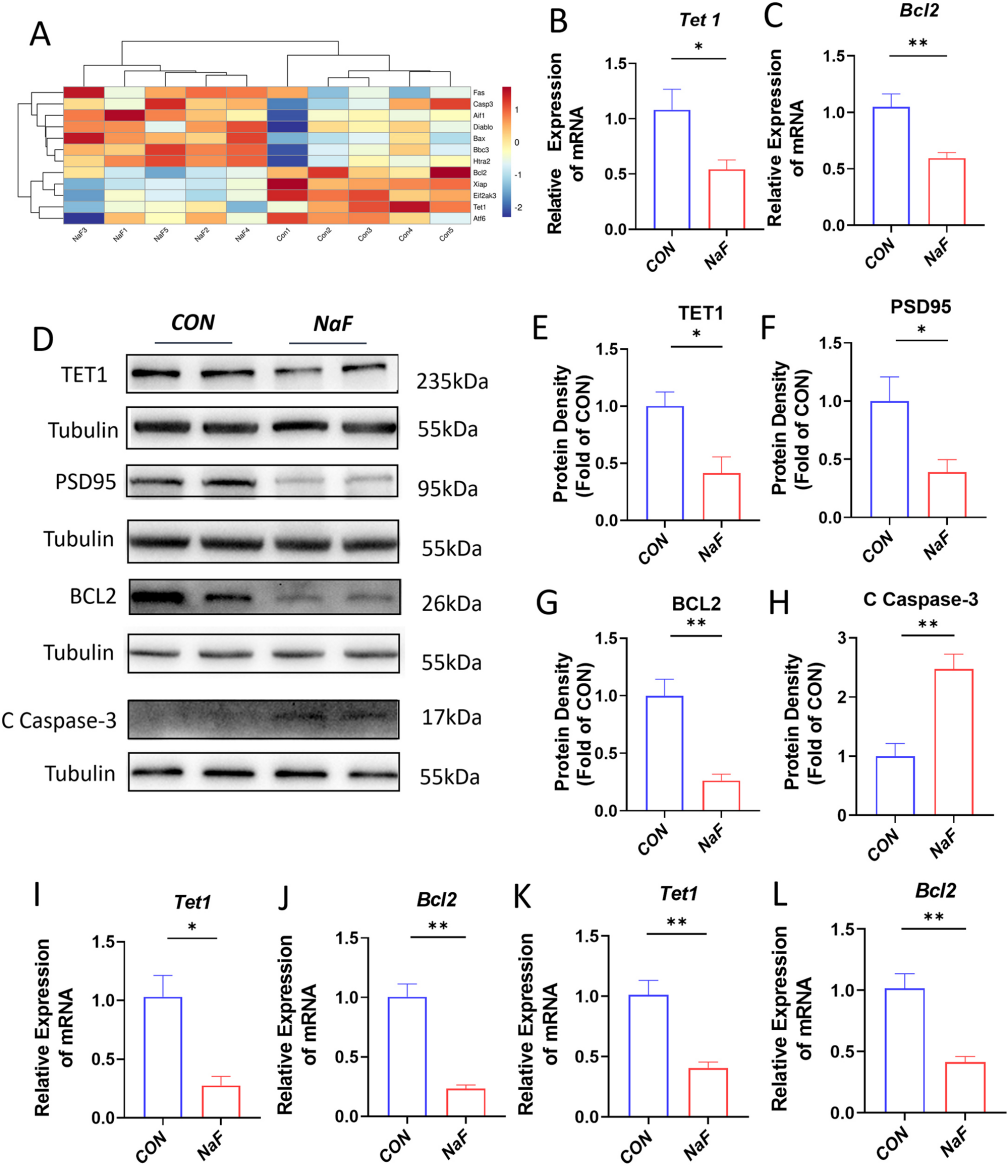
Gene expression characteristics of hippocampal neurons in NaF-exposed mouse offspring (**A**) A heatmap representing differentially expressed genes (DEGs) related to apoptosis. (**B**, **C**) Quantitative real-time PCR analyses of *Tet1* and *Bcl2* in adult mice hippocampus (*n* = 12). (**D**-**H**) Western blot analysis of selected molecules TET1, PSD95, BCL2 and C Caspase-3 in the adult hippocampus from hippocampus in NaF-exposed mouse offspring. Tubulin served as a loading control (*n* = 8). (**I**, **J**) The mRNA level of *Tet1* and *bcl2* in primary hippocampal neurons and (**K** and **L**) prenatal mice hippocampus from NaF-exposed mice (*n* = 6). Data are shown as mean ± SEM. *p < 0.05, **p < 0.01 compared to the corresponding controls

To systematically elucidate the underlying molecular mechanisms, we conducted transcriptome profiling using RNA-seq, and analyzed the alterations in gene expression between hippocampal neurons of NaF-treated and control mice. As shown in Fig. 2A, the corresponding heat map demonstrated that numerous genes were upregulated or downregulated in expression levels in the hippocampus NaF-treated male mice, highly distinguished from and control hippocampus. In general, epigenetic modifications mediate the interactions between gene and environment. The RNA-seq heat map indicated that exposure to a high concentration of fluoride significantly reduced the expression of *Tet1* and certain anti-apoptotic proteins, while significantly increasing the expression of myriad apoptosis protein in male mouse hippocampus. In line with this, quantitative real-time PCR assay confirmed that two representative genes, *Tet1* and *Bcl2*, were significantly downregulated under high fluoride exposure (Fig. 2B and C). Next, western blot analysis further revealed that prenatal exposure to high levels of fluoride resulted in a reduction in the expression of TET1, BCL2 and PSD95, while concurrently increasing the expression of the C-Caspase-3 in the hippocampus of adult male offspring from both experimental groups (Fig. 2D). Quantitative analysis indicated that the expression levels of TET1, BCL2 and PSD95 were significantly decreased by approximately 2- to 3.2-fold in the prenatal high fluoride exposure group compared to the control group, whereas the expression level of C - Caspase-3 was significantly elevated (Fig. 2E and H). Consistently, the expression of these genes was also reduced in both primary hippocampal neurons and E18 embryonic hippocampus treated with NaF (Fig. 2I and L).

### The effect of prenatal fluoride exposure on expression of *Tet1* and apoptosis-related proteins in primary neurons

**Fig. 3:**
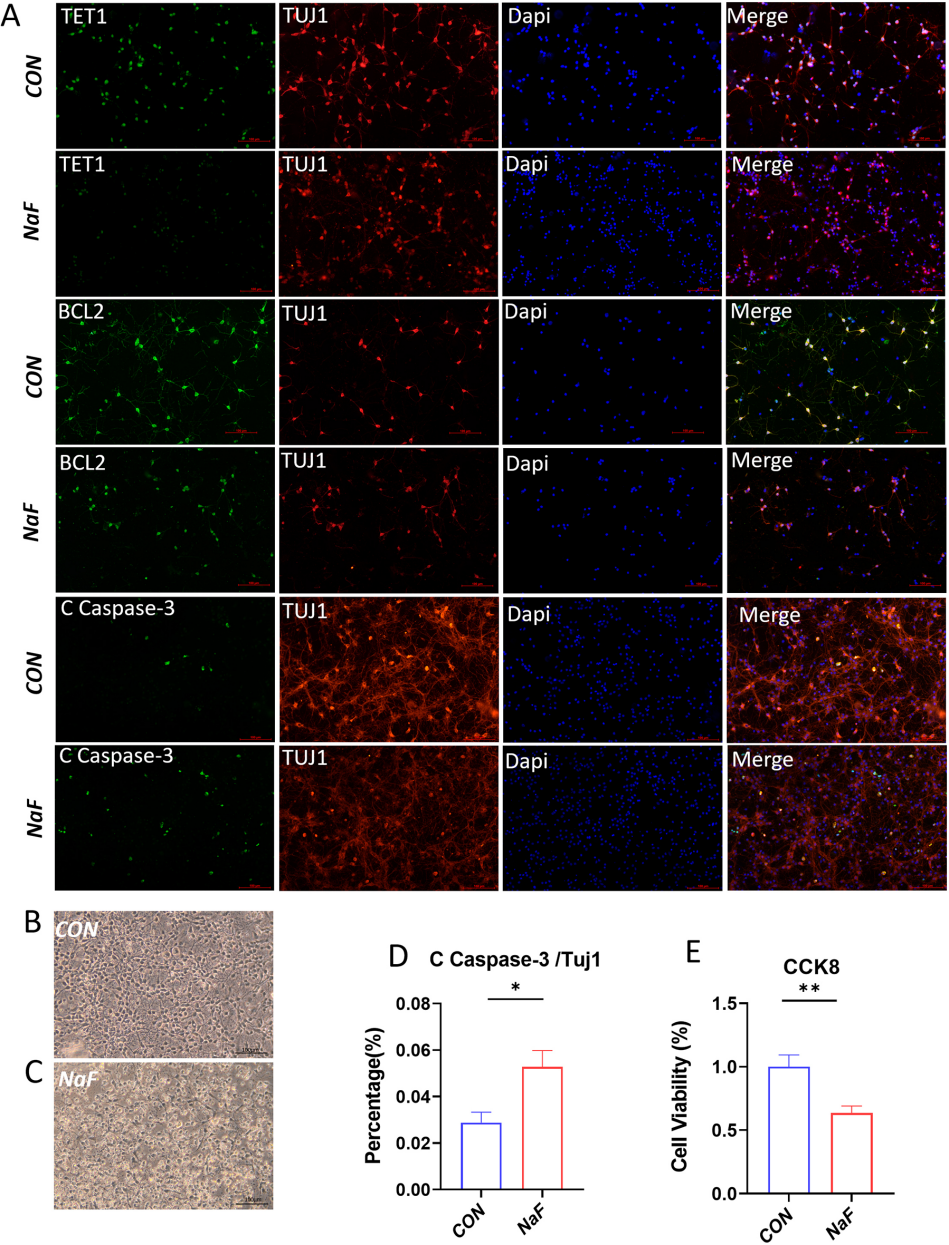
Morphological and biochemical characteristics of primary neurons exposed to high sodium fluoride (**A**) Immunofluorescence analysis of TUJ1, TET1, BCL2 and C-Caspase-3 in primary neuronal cells treated with 0.2mM NaF for 4 days. The overlaid images show TUJ1^+^ (red) neurons, Dapi-stained (blue) nuclei and indicated protein including TET1, BCL2 and C Caspase-3 (green) respectively. (**B**, **C**) Representative Phase-contrast images of primary neurons treated with or without NaF at 4 days in vitro. (**D**) Quantitative assessment of C-Caspase-3 positive neurons under the indicated treatment (*n* = 6). **p < 0.05 vs. the control. (**E**) Quantification of neuron viability via the CCK8 assay revealed that NaF significantly reduced cell viability compared to the control group (Con) (*n* = 12). Data are presented as mean ± SEM. *p < 0.05, **p < 0.01

To further substantiate the hypothesis that prenatal fluoride exposure significantly contributes to the reduction of the intracellular TET1 protein levels, thereby leading to cell apoptosis, we conducted parallel immunofluorescence analyses of TET1, as well as apoptosis-related proteins BCL2 and C-Caspase-3. Consistent with the findings from qPCR and western blot analyses, the double immunofluorescence results for TUJ1 and TET1, as well as BCL2 and C-Caspase-3, demonstrated that the majority of TUJ1-positive cells in the control group exhibited strong immunoreactivity for TET1 and BCL2, with minimal co-localization of TUJ1 and cleaved Caspase-3. In contrast, cells exposed to fluoride showed increased reactivity towards cleaved Caspase-3, an absence of notable TET1 immunostaining, and a significant reduction in BCL2 immunoreactivity among TUJ1-positive cells (Fig. 3A). In addition, exposure of cells to fluoride obviously decreased immunoreactivity of TUJ1 and resulted in increased cellular debris. Morphologically, almost all primary hippocampal neurons in vitro for 4 days exhibited intact cell bodies with a complex neurite network. In contrast, those exposed to 0.2mM NaF for the same duration showed marked morphological alterations including somatic shrinkage, degeneration, and neurite rupture, predominantly characterized by an abundance of necrotic debris and reduced number of viable cells in the culture (Fig. 3B and C). Moreover, quantitative analysis of C - Caspase-3 positive cells also indicated that NaF treatment elicited significantly higher percentage of apoptotic cells compared to the control (Fig. 3D). Consistently, NaF significantly reduced neuronal viability relative to the untreated control group (Fig. 3E). These results suggest that fluoride exposure substantially results in neuronal apoptosis.

### The effects of fluoride exposure on neuron excitability and apoptosis

**Fig. 4:**
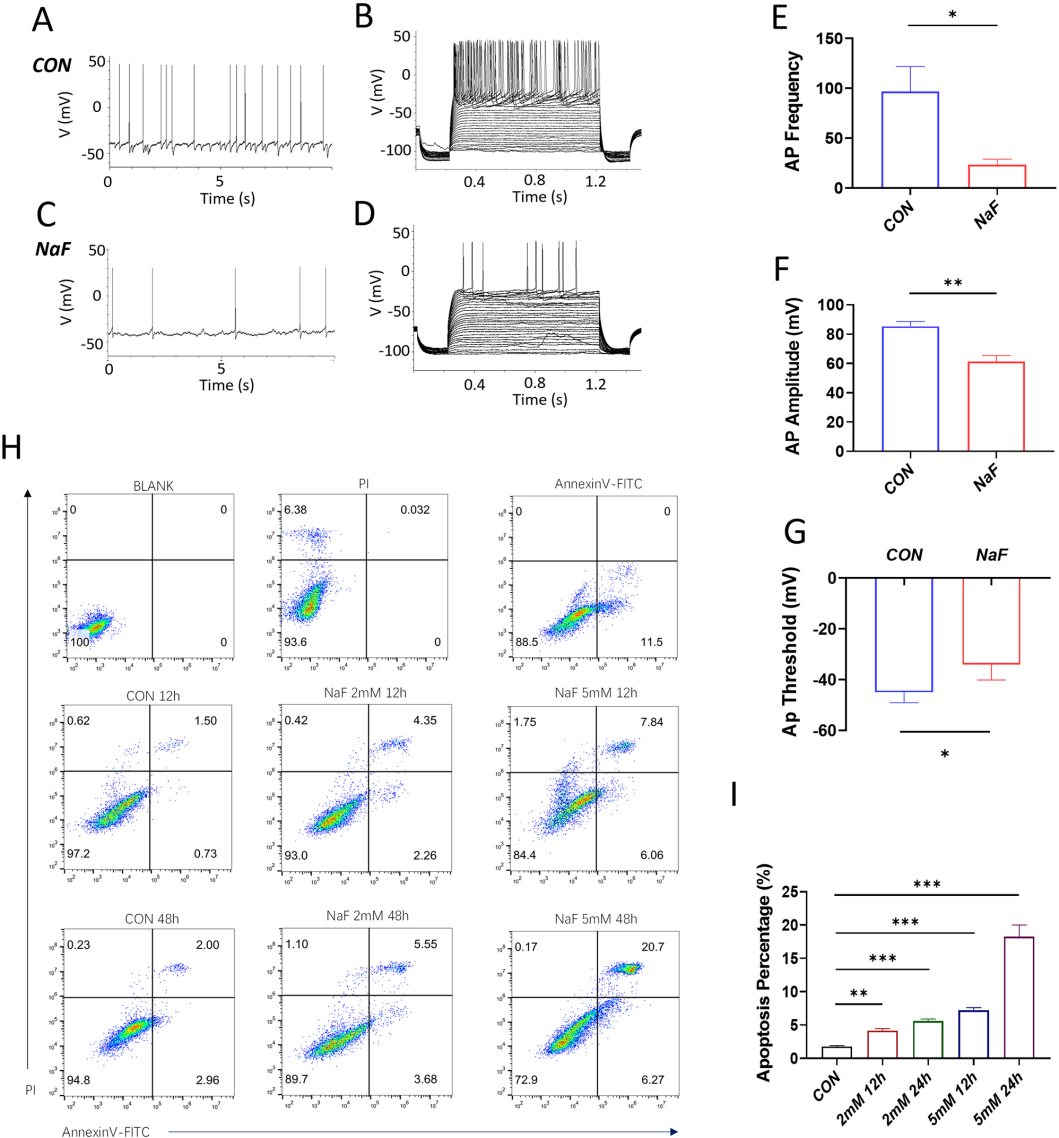
Fluoride exposure reduces neuronal excitability and promotes neuron apoptosis (**A** and **C**) Representative traces of spontaneous AP evoked at 0 pA for fluoride exposure and control, respectively. (**B** and **D**) Traces illustrating APs evoked at different current injections for fluoride exposure and control conditions, respectively. Notably, current injections were performed from − 100 pA to 200 pA with 10 pA increasing step, and lasted for 1s. (**E**) The total frequency of AP evoked by each current injection. Initial point of AP upstroke phase is the threshold (*n* = 7). (**F** and **G**) Neural AP amplitude and threshold measured from the first AP evoked during current injections for the indicated treatment groups (*n* = 7). (**H**) Flow cytometry analysis of apoptotic and viable HT22 cells subjected to the indicated treatments. Notably, the four quadrants represent the percentages of live, apoptotic, late apoptotic and necrotic cells undergoing the indicated treatments, respectively. (**I**) Quantification of the relative number of apoptotic cells undergoing the indicated treatments (*n* = 6). All data are reported as the means ± SEM. Statistical significance is indicated as *p < 0.05, **p < 0.01 and ***p < 0.001 vs. their respective controls

Next, patch clamp and flow cytometry were carried out to further detect the adverse effects of fluoride exposure on hippocampal neurons. As shown in Fig. 4, the NaF-treated neurons exhibited a higher Ap threshold, along with a reduced frequency of spikes and diminished AP amplitude in both spontaneous action potential and in response to intracellular depolarizations, compared to the control group, indicating that fluoride exposure compromised the excitability of neurons (Fig. 4A and G). In addition, the flow cytometric analysis revealed a significant increase percentage of early and late apoptosis in NaF-treated HT22 cells in a time- and dose-dependent manner, as demonstrated by Annexin-V-FITC/PI double staining. Conversely, cell populations in the lower left quadrant were clearly reduced (Fig. 4H). Notably, the percentage of cell apoptosis was significantly enhanced following NaF treatment for 12 h and 48 h at various doses compared to the corresponding control groups (Fig. 4I, **p < 0.01, ***p < 0.001). Collectively, these data suggest that Fluoride exposure can markedly promote the cell apoptosis and necrosis.

### The influence of *Tet1* deficiency and fluoride exposure on morphological changes and cytoarchitecture of hippocampus

**Fig. 5:**
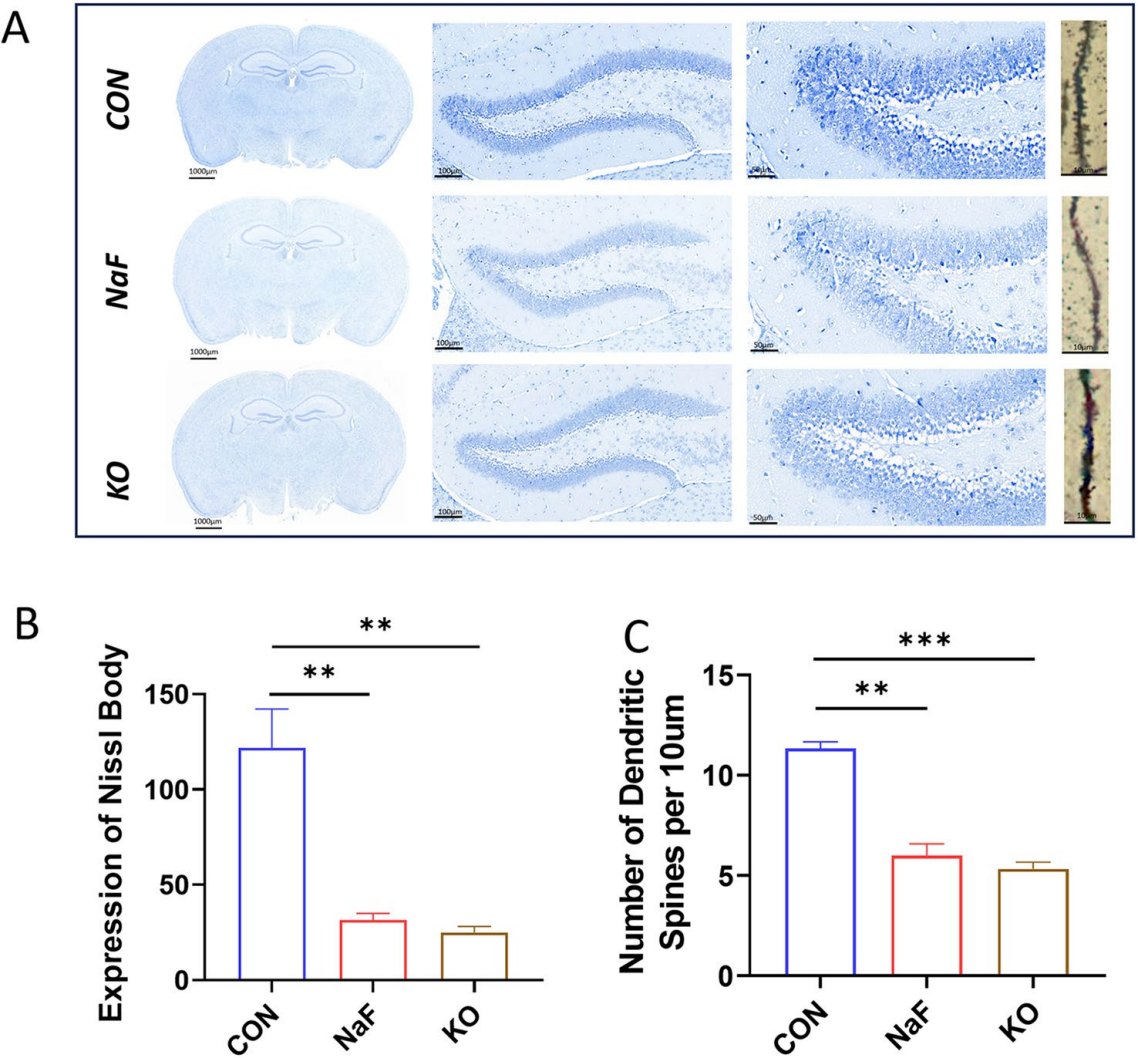
Changes in the morphology and cytoarchitecture of hippocampus in NaF-exposed or *Tet1* deficient mice (**A**) Prenatal exposure to NaF and *Tet1* deficiency induce changes in morphology and cytoarchitecture in male mouse offspring by Nissl staining and Golgi staining. Representative images revealed that both NaF exposure or *Tet1* deficiency all lead to a significant decrease in the number of Nissl-stained neurons and dendritic spines in the hippocampal DG subregion of mice. (**B**) Quantitative analysis of Nissl body expression (*n* = 12), and (**C**) Quantitative assessment of dendritic spine number under the indicated treatments (*n* = 9) show statistically significant differences, with **p < 0.01; ***p < 0.001 compared to their respective controls

To further investigate the biological function of TET1 in prenatal fluoride-induced cognitive impairment, we examined the morphological changes of hippocampus in NaF and *Tet1* deficient male mice using Nissl-staining, Golgi staining, and analyses of cell density and cytoarchitecture. As shown in Fig. 5A, Nissl-stained neurons in the hippocampal subregions of normal mice exhibited clearly visible cell bodies with a polygonal shape, while Golgi staining revealed numerous dendritic spines in the hippocampal DG subregion. In contrast, no significant differences in the Nissl-stained cytoarchitecture and Golgi staining were detectable between NaF-treated and *Tet1*-deficient male mice, despite both groups displaying weaker Nissl-staining and a reduced dendritic spines compared to normal groups. In addition, there was a significant decrease in the number of Nissl-stained neurons and dendritic spines in the hippocampal DG subregion of the two mice relative to the control group (Fig. 5). Notably, *Tet1*-deficient male homozygous mice did not exhibit any lethal morphological and cytoarchitecture abnormalities (Fig. 5A). Although no lethal morphological abnormality appeared, a reduction in Nissl substance and dendritic spines were observed in the hippocampal subregions of *Tet1*-deficient homozygous mice (Fig. 5B and C). These data presented above suggests that *Tet1* deficiency may constitute a significant risk factor as fluoride for spatial learning and short-term memory impairments.

### *Tet1* deficiency compromises mice spatial learning and memory

**Fig. 6:**
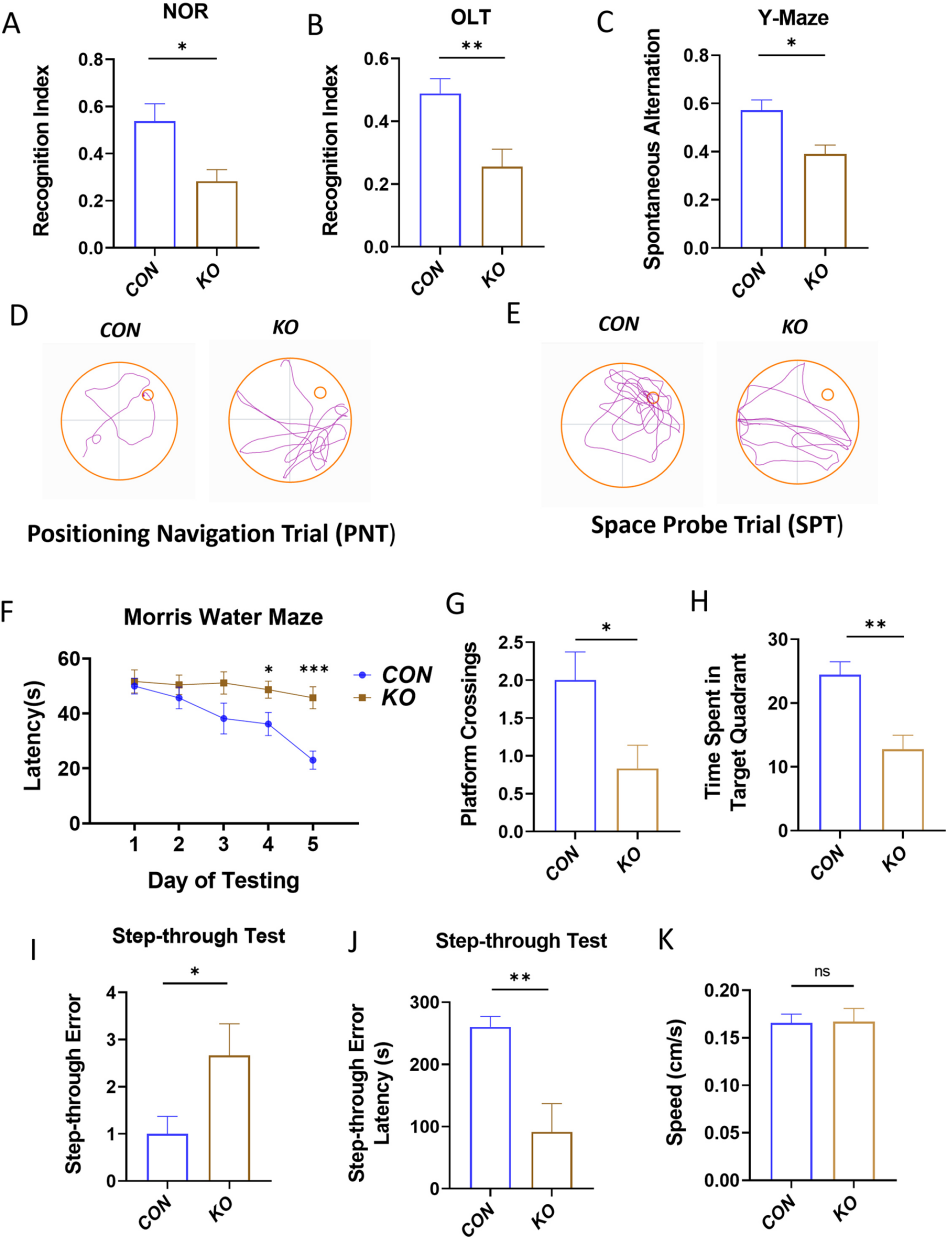
*Tet1* KO compromises mice spatial learning and memory. (**A**) NOR, (**B**) OLT, (**C**) Y-Maze test (*n* = 15). (**D**, **E**) Representative pathway maps of PNT and SPT in Morris water maze test. (**F**) The escape latency of adult offspring from KO mice was measured using a hidden platform (*n* = 15). (**G**) The number of platform crossing of KO mice in Morris water maze test (*n* = 15). (**H**) The duration spent in the target quadrant of KO mice in SPT (*n* = 15). (**I**, **J**) The number of errors and the latency before initially entering the dark chamber in passive avoidance test by KO mice (*n* = 15). (**K**) The swimming speed of KO mice was evaluated (*n* = 15). Notably, no significant difference in Morris water maze test was found between two groups. All data are presented as the means ± SEM. with significance levels indicated as *p < 0.05, **p < 0.01 and ***p < 0.001 compared to the corresponding controls

To investigate the role of Tet1-mediated DNA hydroxylation in the regulation of neural plasticity, we examined 2-month-old *Tet1* whole-body knockout male mice and wild type (WT) mice learning and memory by means of a series of behavioral tests including Morris water maze, NOR, Y-maze and passive avoidance test. As shown in Fig. 6, *Tet1* KO mutant male mice showed a significant decrease in recognition index for both NOR and OLT, as well as spontaneous alternation behavior as compared to the control mice (Fig. 6A, B and C). Of interest, similar to the NaF mice, the Tet1 KO group demonstrated significant deficiencies in short-term memory retention, as evidenced by escape latency, platform crossings, and the time spent in the target quadrant in Morris water maze (Fig. 6D and H). Consistently, passive avoidance test also revealed a significant decrease in spatial and learning memory in *Tet1* KO animals. Likewise, the KO mice also showed a higher step-through errors and reduced latency of step in step-through probe test compared to WT mice (Fig. 6I and J). Nevertheless, *Tet1* KO mutants presented the similar swimming speed as WT mice (Fig. 6K), suggesting that *Tet1* deficiency had no influence on the animals’ motivation. These aforementioned data imply that *Tet1* deficiency may be a significant risk factor for impairments in spatial learning and short-term memory.

### TET1 directly regulates the expression of *Bcl2* by modulating DNA hydroxymethylation of *Bcl2* promoter

**Fig. 7:**
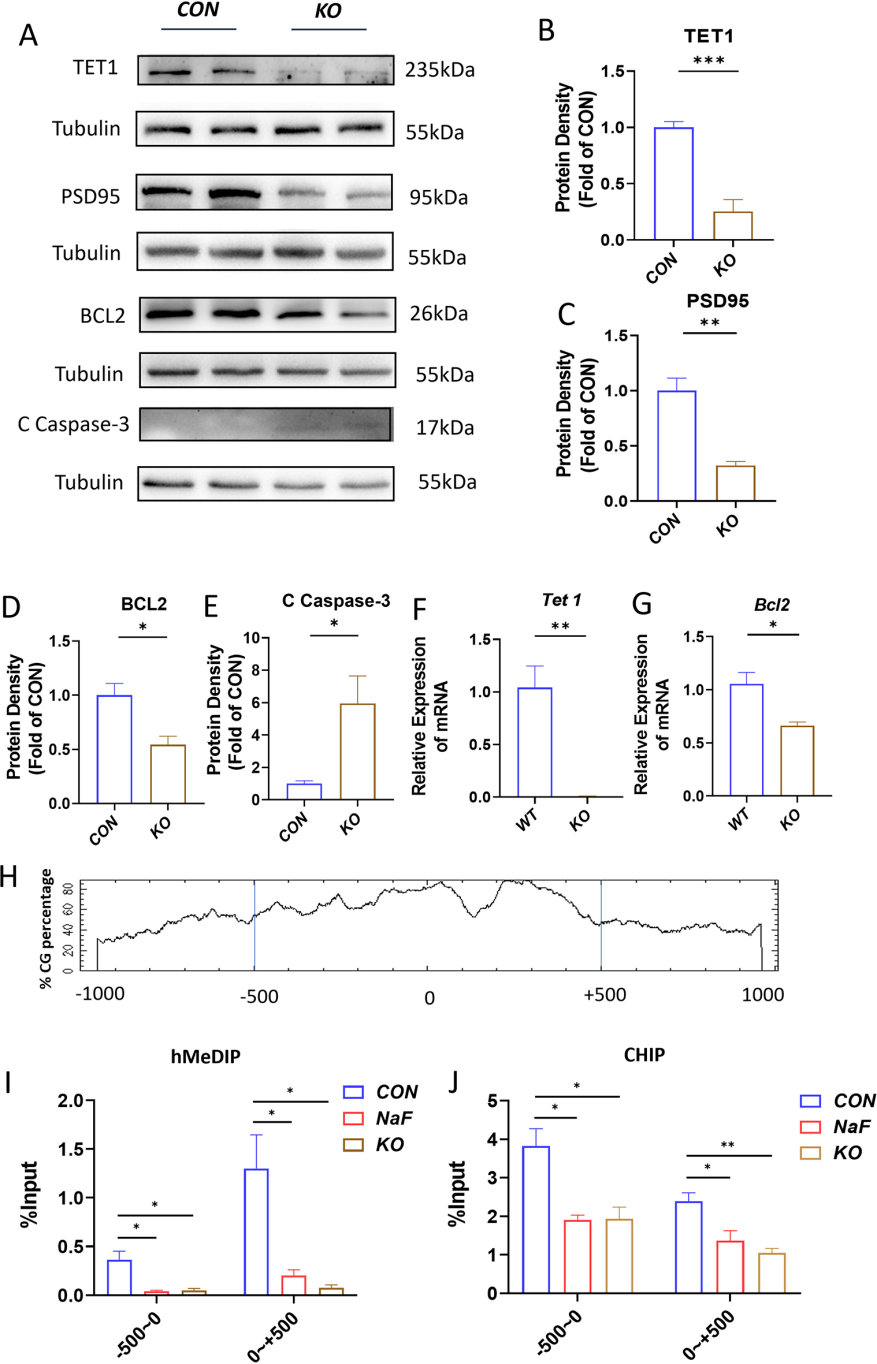
TET1 directly modulates the expression of BCL2. (**A**-**E**) Western blot and quantitative analysis of TET1, PSD95, BCL2 and C-Caspase-3 in the adult hippocampus of *Tet1* deficient mice (*n* = 8). (**F**, **G**) Quantitative real-time PCR analysis of the indicated genes in KO mice (*n* = 6). (**H**) A bioinformatic analysis of CpG islands of *Bcl2* from upstream + 1 kb to downstream − 1 kb region. (**I**) The hydroxymethylation status of *Bcl2* gene promoter CpG islands in adult hippocampus from three groups (*n* = 9). (**J**) ChIP assays were performed to detect TET1 occupancy in *Bcl2* promoter region (*n* = 9). All data are reported as the means ± SEM. *p < 0.05, **p < 0.01. Statistical significance is indicated as *p < 0.05 and **p < 0.01

To further investigate whether TET1 was involved in transcriptional activation through enrichment to the promoter regions of *Bcl2*, the hMeDIP and ChIP assays were conducted to evaluate the DNA hydroxymethylation of *Bcl2* and TET1 protein occupancy within *Bcl2* promotor regions. In accord with findings in the NaF-treated mice group, western blot analysis revealed a significant decrease in the expression of BCL2 and PSD95, alongside a significant increase in the level of C-Caspase-3 in *Tet1* deficient male mice. This was accompanied by a reduced number of dendritic spines and Nissl bodies, indicating a marked involvement of *Tet1* in modulating the neuronal apoptosis (Fig. 7A and G). Thus, one plausible explanation for the impaired spatial learning and memory observed in NaF-treated male mice is the reduced expression of TET1 protein and the heightened rate of apoptosis. To further substantiate the claim that TET1 is likely to regulate the mRNA abundance of *Bcl2*, ChIP-qPCR and hMeDIP were utilized to elucidate the precise mechanism by which TET1 influences *Bcl2* expression. As our expected, sequence analysis of DNA hydroxymethylation of CpG promoter islands revealed a higher CG content from the upstream + 500b to the downstream − 500 kb region (Fig. 7H). In addition, hMeDIP revealed reduced hydroxymethylation ratios from upstream + 500b to downstream − 500 kb region in the *Tet1* KO groups compared to the control group, which is similar to NaF-treated group (Fig. 7I). ChIP-qPCR results also demonstrated a decreased occupancy of TET1 occupy from the upstream + 500 kb to downstream − 500 kb region in both the NaF and *Tet1* KO groups (Fig. 7J). These results suggests that the TET1 protein directly binds to the promotor region of *Bcl2*, modulating its hydroxymethylation and thereby influencing *Bcl2* expression.

## Discussion

Water is one of the most essential resources required for the sustenance of life on the world. Groundwater often constitutes one of the highly extracted water resources that provide drinking water to approximately half of the global population (Abascal et al. 2022; Liu et al. 2022; Thaw et al. 2022). Once anthropogenic environmental damage occurs due to intense human activities, groundwater will be contaminated with heavy metals, metalloids, and non-metals, which severely threatens the health of a large number of people worldwide (Liu et al. 2024; Mintenig et al. 2019; Thomas et al. 2021). Among these contaminants, ground water is particularly susceptible to fluoride contamination due to the widespread presence of fluoride in rocks and minerals (Solanki et al. 2022). Notably, the concentration of fluoride in industrial effluents can range from 250 mg/L to 1500 mg/L, and may even reach levels as high as 10,000 mg/L (Bagastyo et al. 2017). Fluoride accumulation in humans typically occurs through exposure to contaminated water, soil, and biota, which poses serious health risks (Kusrini et al. 2015). Given that fluorosis has emerged as a serious public health concern in many countries, groundwater pollution by fluoride is a primary global issue due to its high toxicity (Adkins et al. 2022; Rango et al. 2014; Ren et al. 2022; Tang et al. 2023). Currently, extensive studies have demonstrated that exposure of rodents to high fluoride environments can trigger animal behavioral and cognitive deficits (Ge et al. 2018; Yuan et al. 2019; Zhao et al. 2019). Although a growing number of studies have substantiated that fluorosis can impair brain functions through oxidative stress, mitochondrial structural damage, metabolic abnormalities, and interference with antioxidant enzyme activity (Zhou et al. 2020), little is known about the molecular mechanisms underlying of excessive fluoride exposure-induced cognitive deficiencies in the adult offspring.

Accumulating previous studies have indicated that excessive fluoride exposure leads to deficits in intelligence, memory and cognition in both humans and rodents (Choi et al. 2012; Tang et al. 2024). In line with these finding, our latest results demonstrated that fluoride exposure during pregnancy compromises neurodevelopment and results in learning and memory impairments in adult male offspring, without a discernible impact on motor abilities (Li et al. 2022). A NOR test was conducted on female offspring, revealed that while NaF exposure affected female mice to some extent, the impact was considerably less pronounced than in males (Figure S1C). This preliminary observation suggests a potential neuroprotective role of estrogen in the CNS. The existing literature indicates that estrogen receptors (ERs) are widely expressed in brain regions involved in learning and memory, including the prefrontal cortex, hippocampus, amygdala, cingulate cortex, and retrosplenial cortex. Estrogen, through these receptors, regulates critical signaling and transcriptional pathways, thereby facilitating neuronal energy production to maintain cellular function and ensuring a rapid and efficient neural response (Brinton et al. 2015). To eliminate the potential confounding effects of estrogen, the study focused predominantly on male offspring. Furthermore, fluoride induces neuronal death, morphological alterations and electrophysiology in hippocampus partially by the regulation of epigenetic modifications. These results substantiate the notion of the hippocampal long-term cognition deficiencies arising from prenatal fluoride exposure. As a result, the urgent need for elucidating the underlying mechanisms to antagonize this damage may be of vital importance.

In the present study, we found fluoride exposure during pregnancy largely results in wide array of cognitive deficiency. This exposure is associated with decreased the expression of TET1, reduced the density of dendritic spine, and diminished PSD95 expression in the hippocampus of the adult offspring. Molecular and cellular evidence further demonstrated that fluoride treatment remarkably exacerbates neuronal apoptosis and decreases neuronal excitability by dramatically inhibiting the expressions of anti-apoptotic protein BCL2 and downregulating TET1. More importantly, TET1 appears to regulate *Bcl2* expression by directly binding to the promotor region of *Bcl2* and enhancing its DNA hydroxymethylation levels. In addition, ChIP-qPCR and hMeDIP assays provided direct evidence that the reduced BCL2 expression in the hippocampus of fluoride-treated mice is attributable to the change of 5hmC level around the TSS of *Bcl2*. Therefore, the present comprehensive mechanistic study elucidating the involvement of TET1 in modulating BCL2 expression against neural apoptosis through increasing DNA hydroxymethylation will provide a novel therapeutic target for addressing prenatal fluoride-induced cognitive impairments.

The stability of the hippocampal internal environment across various developmental stages is of vital importance for the learning and memory function (Agnihotri et al. 2004). Once disruption of hippocampal tissue structure and cytoarchitecture, and synaptic molecular composition by a variety of insults such as chronic inflammatory, oxidative stress, and neurotoxin can lead to the cognitive function decline (Du et al. 2022). Our present study demonstrates that high fluoride exposure can markedly cause neurocognitive impairment, displaying the cognitive and spatial memory deficits of male offspring. These findings are in line with our recent reports (Li, Lu, Zhu, Liu, Shi, Zeng, Yu, Guo, Wei, Cai and Sun 2022). This result suggests that fluoride may damage hippocampal cytoarchitecture and even potentially affect SGZ neurons responsible for cognition and memory. For elucidation of our hypothesis that the cognitive and spatial memory deficits in offspring arising from high fluoride exposure are likely due to cell apoptosis or necrosis in the hippocampus, we further detected NaF-exposed neuronal apoptosis. Intriguingly, the present results demonstrates that the expression of C-Caspase-3 was significantly elevated, while the expression of *Bcl2* significantly reduced. In line with the results of neuronal apoptosis, CCK8 assays further confirmed the cytotoxicity of fluoride on neurons. In addition, the expression of PSD95 and TET1 were markedly decreased. These findings suggest that neuronal apoptosis is one of the contributing factors to cognitive impairment in male offspring following prenatal sodium fluoride exposure, as evidenced by the significant changes in the expression levels of apoptosis-related proteins BCL2 and C-Caspase-3, which act as mediator or effector. However, other cognition-related mechanisms, such as oxidative stress, calcium overload, and mitochondrial dysfunction, remain intriguing and warrant further investigation. Coincidentally, PSD95, a postsynaptic density protein associated with in memory and learning, also exhibited decreased expression (Bustos et al. 2017). Importantly, a significant reduction in the neuronal excitability was observed in neurons treated with NaF, indicating that fluoride exposure-induced neuronal apoptosis likely contributes to the cognitive and spatial memory deficits of offspring.

Despite the established link between fluoride-induced neuronal apoptosis and cognitive and spatial memory deficits in offspring, it remains unclear whether *Tet1* KO produces outcomes similar to fluoride exposure. Consequently, we investigated the relationship between TET1 and the anti-apoptotic protein BCL2. Reportedly, epigenetic modifications are widely recognized as mediators of gene-environment interactions (Wu et al. 2023). DNA methylation and hydroxymethylation are crucial in regulating gene expression (Yan et al. 2023). In the study, we found that prenatal fluoride exposure can cause a significant decrease of *Bcl2* expression, which is similar to the effects observed in *Tet1* KO mice, implying the involvement of TET1 protein in modulating *Bcl2* expression. Given that DNA methyltransferases and demethylases collaboratively regulate the levels of 5-methylcytosine (5mC) and 5-cytosine (5 C), with DNA methylation indirectly suppressing DNA transcription through effecting the binding of the methyl-CpG binding protein (MeCP1) (Cross et al. 1997; Guo et al. 2017). Importantly, our ChIP-qPCR and hMeDIP assays further provided direct evidence that the reduced expression of BCL2 in the hippocampus of fluoride-treated mice was attributable to alterations in the 5hmC level around the TSS of *Bcl2*. On the basis of our present bioinformatic analysis of CpG islands of *Bcl2* in combination with sequential oxidation of 5-mC to 5-hydroxymethylcytosine (5-hmC) by TET enzymes, we speculated that TET1 mitigates prenatal fluoride-induced cognitive impairment by modulating the DNA hydroxymethylation of *Bcl2*, leading to its up-regulation. Intriguingly, *Tet1* knockout mice also showed an identical abnormality in hippocampal morphology and cytoarchitecture responsible for the spatial learning and memory, resulting in cognitive deficits. Based on experimental findings from KO mice, even in the absence of NaF exposure, these mice exhibited phenotypes and molecular mechanisms similar to those observed in NaF-exposed mice. This observation suggests that the TET1-mediated regulation of BCL2 expression may not be exclusively linked to embryonic NaF-induced neurotoxicity but may instead play a broader role in neuronal development and regulation. Furthermore, the present study revealed that the TET1 protein also played a positive role in the epigenetic regulation of the adult brain. Similarly, recent study has demonstrated that TET1 along with early growth response 1 (EGR1) can remove persistent methylation marks in the brain during developmental life experiences, thereby activating downstream genes (Sun et al. 2019). Additionally, studies have demonstrated a link between TET1 and adult hippocampal neurogenesis and pain hypersensitivity (Hsieh et al. 2017; Zhang et al. 2013). Based on our current data in combination with existing literatures, it is plausible that TET1 protein deficiency and demethylation disorders of the *Bcl2* promotor likely contribute to a potential mechanism underlying fluoride elicited cognitive disorders.

Interestingly, TET1 is implicated in the regulation of BCL2, thereby influencing apoptosis, and is also involved in a range of biological processes, including tumorigenesis, myelin regeneration, and oxidative stress (Moyon et al. 2021; Sun et al. 2021; Xin et al. 2015). Studies have shown that *Tet1* KO mice exhibit increased ROS deposition, enhanced fibrosis, severe inflammation, and cell cycle arrest in renal tissues upon exposure to harmful stimuli. Notably, antioxidant treatment with tempol partially mitigated renal injury, with mechanistic studies revealing that TET1 reduces 5mC levels at the promoters of Sod1 and Sod2, thereby enhancing their expression (Fan et al. 2023). Another study demonstrated that when oxidative stress was mitigated by agents increasing antioxidant enzyme levels, the expression of apoptotic markers Bax, C-Caspase-3, and C-Caspase-9 were suppressed, while upregulating the anti-apoptotic protein BCL2 (Gao et al. 2022). This suggests a potential indirect interaction between these molecular pathways, which warrants further investigation. More intriguingly, TET1 deficiency in 5xFAD mice has been associated with increased plaque burden, thereby accelerating the progression of Alzheimer’s disease (Armstrong, Jin, Vattathil, Huang, Schroeder, Bennet, Qin, Wingo and Jin 2023). In conclusion, TET1 plays a therapeutically significant role in multiple disease models, such as acute kidney injury and Alzheimer’s disease, highlighting its notable research value and broad clinical application potential.

In forthcoming studies, we intend to administer NaF exposure in Tet1 KO animals. Should cognitive function remain unchanged, this would further substantiate the essential role of TET proteins in this model. Conversely, if cognitive impairment exacerbates, it would indicate the involvement of alternative proteins or molecular modifications, thereby necessitating further exploration to elucidate additional, yet-undiscovered mechanisms.

## Conclusions

In this study, we found that fluoride can alter the excitability of neurons, decrease the expression of PSD95, and reduce both Nissl bodies and the density of dendritic spines in male offspring hippocampus. Strikingly, the reduced expression of TET1 significantly suppressed the expression of anti-apoptotic protein BCL2 through epigenetic modification, contributing to the neuron apoptosis in the hippocampus of male offspring, which may be one of the underlying mechanisms in the cognitive deficits due to prenatal fluoride exposure. Therefore, these results suggest that *Tet1* may be one of the key molecules involved in neuronal activity and cognitive regulation. Furthermore, TET1 presents a promising therapeutic target for addressing cognitive impairment induced by prenatal sodium fluoride exposure.

## Electronic supplementary material

Below is the link to the electronic supplementary material.

Supplementary Material 1

Supplementary Material 2: Figure S1 Gene targeting strategy (A) Gene targeting strategy. (B) PCR genotyping of mutant mice. Primer locations are indicated in panel A and their sequences are listed in the method. (C) NOR of NaF-exposed mice female offspring. (D) The stimulation current of AP.

## Data Availability

No datasets were generated or analysed during the current study.
