# Supplementary figures and images for: TET1 mitigates prenatal fluoride-induced cognition impairment by modulating Bcl2 DNA hydroxymethylation level
Source: Mol Med. 2025 Mar 25;31:117. doi: 10.1186/s10020-025-01174-w (PMC11938627; doi:10.1186/s10020-025-01174-w)

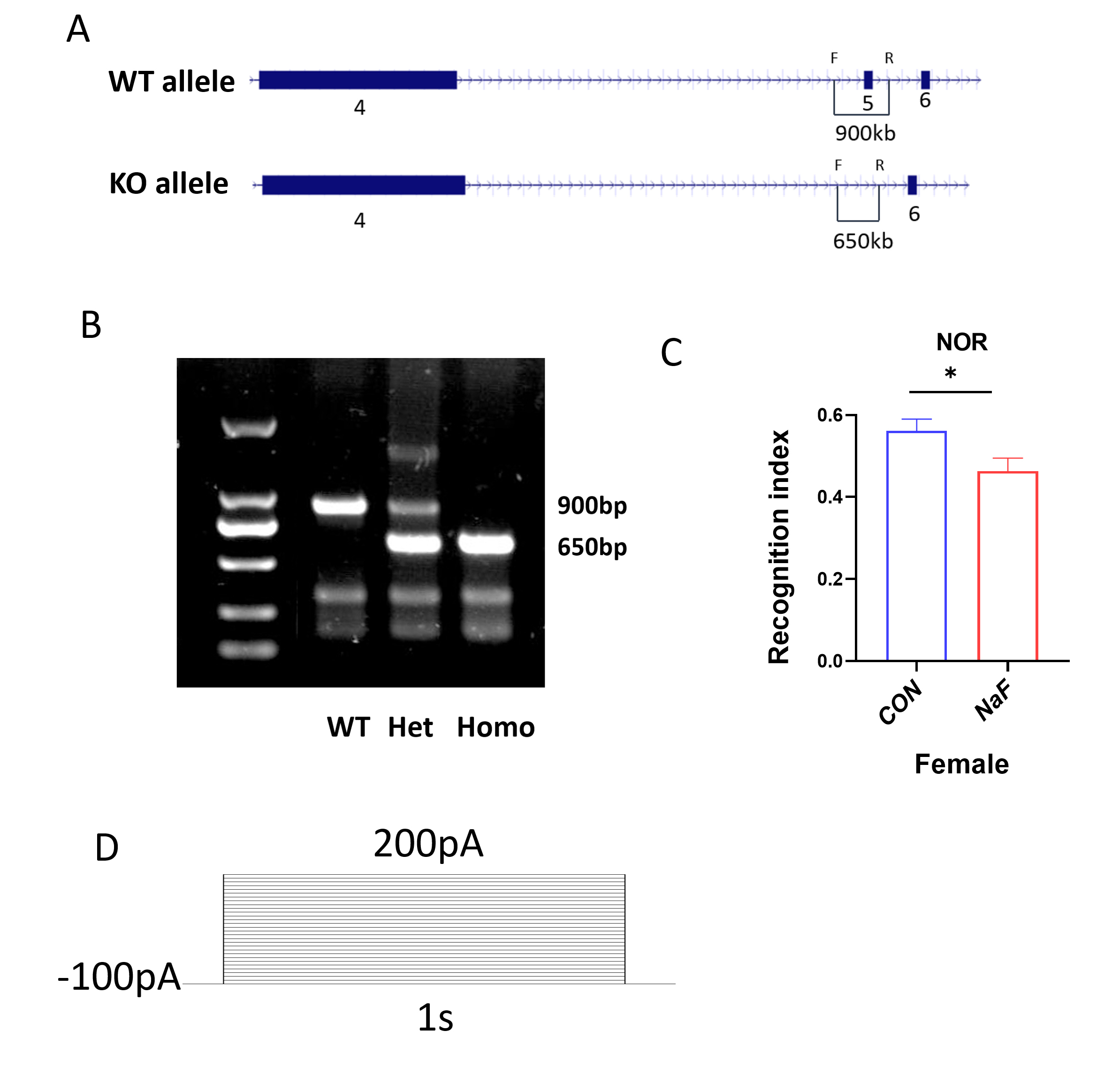

Supplement: Supplementary file 1 — Supplementary Material 1 [file 10020_2025_1174_MOESM1_ESM.jpg]
