# Supplementary material for: TET1 mitigates prenatal fluoride-induced cognition impairment by modulating Bcl2 DNA hydroxymethylation level
Source: Mol Med. 2025 Mar 25;31:117. doi: 10.1186/s10020-025-01174-w (PMC11938627; doi:10.1186/s10020-025-01174-w)
Supplement: Supplementary file 2 — Supplementary Material 2: Figure S1 Gene targeting strategy (A) Gene targeting strategy. (B) PCR genotyping of mutant mice. Primer locations are indicated in panel A and their sequences are listed in the method. (C) NOR of NaF-exposed mice female offspring. (D) The stimulation current of AP. [file 10020_2025_1174_MOESM2_ESM.docx]

Figure S1 Gene targeting strategy (A) Gene targeting strategy. (B) PCR genotyping of mutant mice. Primer locations are indicated in panel A and their sequences are listed in the method. (C) NOR of NaF-exposed mice female offspring. (D) The stimulation current of AP

**Table S1** The sequences of primer sets used for RT-PCR and qPCR analysis.

**Primer sets used for RT-PCR and qPCR**

| **Gene** | **Forward primer** | **Reverse primer** |
| --- | --- | --- |
| ***Gapdh*** | **5’-CATGGCCTTCCGTGTTCCTA-3’** | **5’-CTTCACCACCTTCTTGATGTCATC-3’** |
| ***Tet1*** | **5’-AGCTCATGGAGACTAGGTTTGG-3’** | **5’-TGGTCTACACGCTCACGAAC-3’** |
| ***Bcl2*** | **5’-GGATGACTTCTCTCGTCGCT-3’** | **5’-GACGCTCTCCACACACATGA-3’** |
| ***Bcl2* 0 ~ + 500** | **5’-TGCTGGCTGGACGTGCCTATA-3’** | **5’-TCCATGCAATTGTGCCCATCCT-3’** |
| ***Bcl2* -500 ~ 0** | **5’-GCTAAATGCAGGCACGGACG-3’** | **5’-GGGACTTCGTAGCAGTCATCCT-3’** |
